# Functional and ecological characterization of *Labrys methylaminiphilus* subsp. *lupini* subsp. nov., associated with *Lupinus luteus* nodules in acidic soils of southern Chile

**DOI:** 10.3389/fmicb.2026.1759558

**Published:** 2026-03-09

**Authors:** Grace Armijo-Godoy, Luis Cottet, Annally Rupayan, Makarena Carrasco, Daniela Levicoy, Haroldo Salvo-Garrido

**Affiliations:** 1 Agriaquaculture Nutritional Genomic Center (CGNA), Temuco, Chile; 2 Escuela de Agronomía, Facultad de Ciencias, Ingeniería y Tecnología, Universidad Mayor, Temuco, Chile; 3 Centro para la Resiliencia, Adaptación y Mitigación (CReAM), Universidad Mayor, Temuco, Chile

**Keywords:** beneficial bacteria, *Labrys methylaminiphilus* subsp. *lupini*, *Lupinus luteus*, rhizosphere, plant-microbe interactions, subspecies, acidic soils

## Abstract

**Background:**

Members of the genus *Labrys* are widely distributed in soil and plant-associated environments, yet their ecological roles and functional contributions within plant-associated microbiomes remain poorly understood. *Labrys methylaminiphilus* strain La1 was isolated from nodules of *Lupinus luteus* growing in acidic soils of southern Chile, providing an opportunity to investigate strain-level traits relevant to plant–microbe interactions under environmental stress.

**Methods:**

Strain La1 was characterized using physiological and biochemical, chemotaxonomic, and genomic approaches, including whole-genome sequencing and comparative genomics. Functional traits related to plant interaction were assessed through *in vitro* assays for indole-3-acetic acid (IAA) production, antifungal activity against lupine pathogens, and in planta experiments evaluating plant growth under salinity and osmotic stress. The ecological distribution of closely related taxa was inferred from screening of publicly available environmental microbiomes using protologger pipeline.

**Results:**

Although strain La1 showed high genomic similarity to *L. methylaminiphilus* JLW10^T^, it exhibited distinct phenotypic, metabolic, and ecological features. These included tolerance to acidic and moderately saline conditions, utilization of rhizosphere-associated carbon sources, and a fatty acid profile consistent with adaptation to terrestrial environments. Genomic analyses revealed genes related to stress tolerance, exopolysaccharide biosynthesis, carbohydrate-active enzymes, siderophore production, IAA synthesis, and non-ribosomal peptide synthetases. Consistent with these traits, La1 inhibited the growth of *Colletotrichum lupini* and *Pleiochaeta setosa* and significantly enhanced *L. luteus* biomass under osmotic and salinity stress. Metagenomic screening indicated that sequences closely related to La1 are predominantly associated with soil, rhizosphere, and plant-associated habitats.

**Conclusion:**

This study demonstrates that strain La1 represents a functionally versatile and ecologically specialized lineage within *L. methylaminiphilus*, contributing traits relevant to plant-associated microbiomes in acidic soils. This integrated functional and ecological evidence supports the designation of *Labrys methylaminiphilus* subsp. *lupini* subsp. nov. and highlights the relevance of strain-level analyses for understanding plant–microbe interactions.

## 1 Introduction

The genus *Labrys* belongs to the class *Alphaproteobacteria*, order *Rhizobiales*. It comprises bacteria with diverse metabolic capabilities and ecological adaptations, including degradation of organic compounds, and associations with plant environments. Members of this genus have been isolated from aquatic sediments, soils and plant-associated habitats, suggesting a broad ecological distribution (Miller et al., 2005; Chou et al., 2007; Carvalho et al., 2008; Tapia-García et al., 2020; Csépányi et al., 2025). Despite this diversity, the functional roles of *Labrys* species within plant-associated microbiomes remain poorly characterized.

Recent advances in microbiome research have highlighted the importance of strain-level resolution for understanding plant–microbe interactions (Choi et al., 2021; Zhang et al., 2023). While community-level surveys provide valuable insights into microbiome composition, they often fail to capture the functional heterogeneity that exists among closely related taxa (Fadiji et al., 2025; Wu et al., 2025). Individual bacterial strains, even within the same species, can differ markedly in traits related to stress tolerance, hormone production, antimicrobial activity, and root colonization, all of which may influence plant performance under environmental constraints (Fitzpatrick et al., 2020). Therefore, detailed functional and ecological characterization of individual strains is increasingly recognized as essential for linking microbiome structure with ecosystem function.

Several *Labrys* species have been reported in association with plants, including *Labrys neptuniae*, isolated from root nodules of the aquatic legume *Neptunia oleracea* (Chou et al., 2007); and *Labrys okinawensis*, recovered from rhizosphere or root-associated environments (Islam et al., 2007; Nguyen et al., 2015; Kim et al., 2020; Chávez-Ramírez et al., 2022). However, in most cases, these reports have focused primarily on taxonomic description, with limited assessment of functional traits relevant to plant interaction or ecological adaptation. As a result, the contribution of *Labrys* lineages to plant health, stress resilience, or rhizosphere competition remains largely unexplored.

Acidic soils represent challenging environments for plant growth due to nutrient limitations, aluminum toxicity, and osmotic stress. In such systems, plant-associated microorganisms can play a critical role in improving plant performance by modulating nutrient availability, producing phytohormones, mitigating stress responses, and suppressing phytopathogens (Msimbira and Smith, 2020). *Lupinus* species, including *Lupinus luteus*, are well adapted to acidic soils and host diverse microbial communities in their rhizosphere and nodules. However, the functional diversity of non-rhizobial bacteria associated with lupine nodules and their potential contributions to plant performance remain insufficiently understood.

In this study, we investigated *Labrys methylaminiphilus* strain La1, isolated from nodules of *L. luteus* growing in acidic soils of southern Chile, as a representative of a plant-associated lineage within the genus *Labrys*. By integrating physiological, biochemical, chemotaxonomic, genomic, and ecological analyses we aimed to (i) determine the taxonomic position of strain La1 within the species *L. methylaminiphilus*, (ii) characterize functional traits relevant to plant–microbe interactions and stress tolerance, and (iii) assess its ecological distribution using metagenomic data from diverse environments. Beyond its taxonomic relevance, strain La1 provides a model to explore how individual bacterial members contribute to plant-associated microbiomes. In low-diversity or stress-prone systems such as acidic soils, strain-level functional characterization is increasingly recognized as essential for linking microbiome composition with ecological function. In this context, the physiological, genomic, and ecological traits of strain La1 allow us to bridge microbial taxonomy with plant–microbe interactions and to highlight the potential relevance of *Labrys* lineages within legume-associated microbiomes.

## 2 Material and methods

### 2.1 Strain Isolation and phenotypic characterization

**FIGURE 1:**
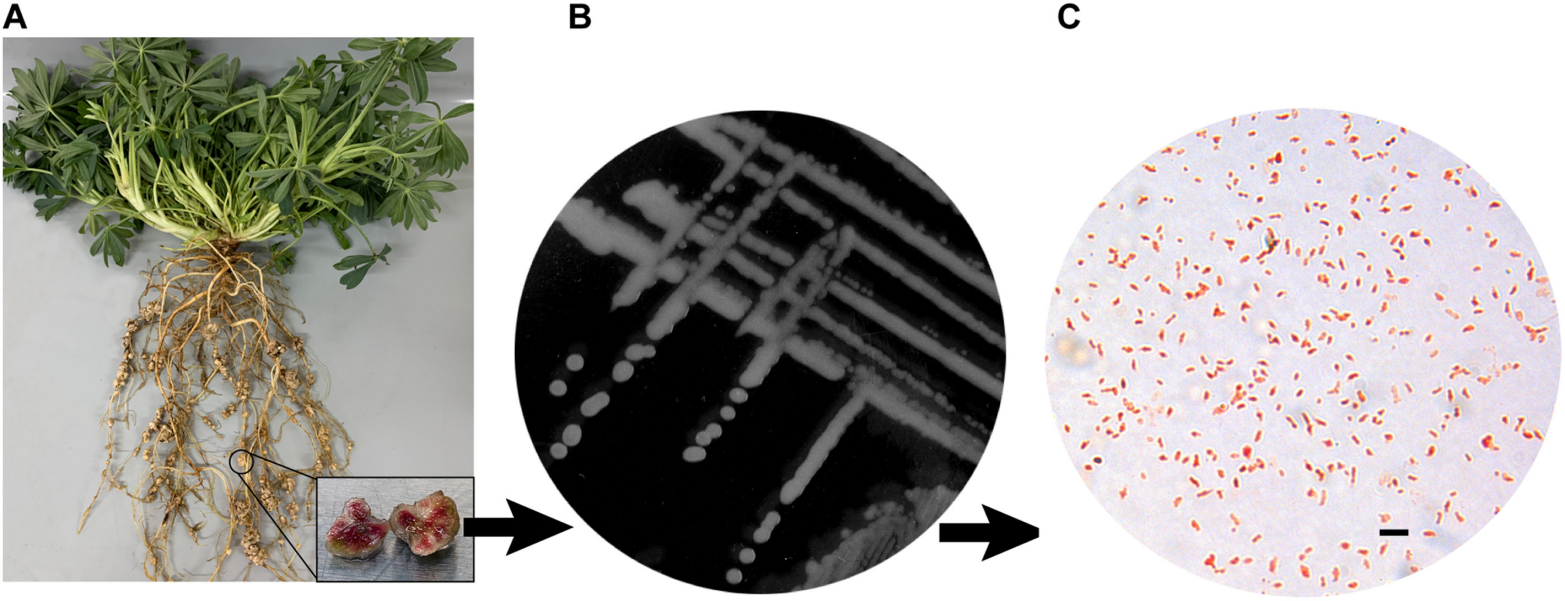
Strain Isolation and phenotypic characterization of *Labrys* La1. **(A)** *L. luteus* Core 98 plant with nodules taken from the field. **(B)** Colonies of *L. lupini on* YMB agar. **(C)** Gram staining analyzed in an optical microscope (×1,000 magnification). Bar represents 1 μm.

*Labrys* strain La1 was isolated from nodules of *Lupinus luteus* L. Core 98 (PI385149), a wild accession of the CGNA’s germplasm collection grown in Vilcún, La Araucanía Region, Chile (38° 41’ 44.04′′ S; 72° 25’ 1.94′′ W). The nodules were separated from the roots (Figure 1A), surface-sterilized with sodium hypochlorite, and washed three times with sterile distilled water. They were then macerated and diluted in a sterile phosphate buffer. Serial dilutions were plated onto yeast-mannitol broth (YMB; yeast extract 1 gL^–1^; mannitol 10 gL^–1^; dipotassium phosphate 0.5 gL^–1^; magnesium sulfate 0.2 gL^–1^; sodium chloride 0.1 gL^–1^ and calcium carbonate 1 gL^–1^) and incubated at 28°C for 48 h to isolate bacterial colonies. For further study, the isolates were purified via repeated subculture on YMB and stored in 50% glycerol at –80°C. Gram staining was performed after culturing cells for 2 days at 28°C on YMB, as described by Tripathi et al. (2025). Cells were analyzed in an optical microscope [Olympus-BX40, Tokyo, Japan (× 1,000 magnification)]. The pH growth tests were performed in Luria Bertani broth (LB; peptone 10 gL-1; yeast extract 5 gL-1; sodium chloride 5 gL-1) adjusted with sterile KOH or HCl solutions to the corresponding pH. The salt tolerance was determined by inoculating the strain in LB supplemented with 0.1–5% (w/v) NaCl.

### 2.2 Phylogenetic analysis

The genomic DNA purification was performed from bacteria cultured for 16 h in LB medium using the Zymo Quick-DNA Fungal/Bacterial Miniprep Kit, according to manufacturer’s instructions. A 16S rRNA gene fragment was amplified by PCR using the 27F (AGAGTTTGATCCTGGCTCAG) and 1492R (GGTTACCTTGTTACGACTT) primers, as previously described (Mendes et al., 2013). The amplicon was sequenced at the Sequencing Center of the Catholic University of Chile. The sequence was assembled and cured using SeqTrace v0.9 (Stucky, 2012), and the bacterial genus was evaluated by pairwise sequence similarity using global alignment through the EzBioCloud server (Yoon et al., 2017a).

For the phylogenetic inference, the 16S rRNA gene sequence corresponding to different species of the *Labrys* genus were obtained from the List of Prokaryotic names with Standing in Nomenclature (LPSN) (Parte et al., 2020). First, multiple sequence alignments were performed using the MAFFT v7.505 program (Katoh and Standley, 2013). Then, the phylogenetic reconstruction based on the 16S rRNA gene sequence was conducted in the MEGA program using the neighbor-joining (NJ) and maximum-likelihood (ML) algorithms (Tamura et al., 2021) inferred using IQ-TREE. The TN93 evolutionary model was used to generate evolutionary distance matrices, and the robustness of the trees was assessed by 1,000 bootstrap replications.

### 2.3 Whole-genome sequencing and genome assembly

The genome of strain La1 was subjected to single-end sequencing using Oxford Nanopore Technologies (ONT), and paired-end sequencing using Illumina MiSeq. In the first case, the libraries were prepared using the PCR-free ONT Ligation Sequencing Kit (SQK-NBD114.24) according to the manufacturer’s specifications, with the NEBNext^®^ Companion Module (E7180L). Nanopore sequencing was conducted on an Oxford Nanopore MinION Mk1B sequencer or a GridION sequencer, utilizing R10.4.1 flow cells in one or more multiplexed shared-flow-cell runs. The run design employed the 400 bp sequencing mode with a minimum read length of 200 bp, and adaptive sampling was not enabled. Illumina sequencing libraries were prepared using the tagmentation-based and PCR-based Illumina DNA Prep kit and custom IDT 10 bp unique dual indices (UDI) with a target insert size of 320 bp. Illumina sequencing was performed on an Illumina NovaSeq 6000 sequencer in one or more multiplexed shared-flow-cell runs, producing 2 × 151 bp paired-end reads. Porechop v0.2.4 was employed to trim residual adapter sequences from ONT reads that may have been overlooked during base-calling and demultiplexing. Demultiplexing, quality control, and adapter trimming were performed with bcl-convert (v4.1.5) for Illumina reads.

*De novo* genome assemblies were generated from ONT read data using Flye v2.9.5 under the nano-hq model, and the options initiate the assembly by first utilizing reads longer than an estimated N50 based on a genome size of 6 Mbp. Subsequent polishing utilized the Illumina read data with Pilon v1.24 under default parameters. To minimize erroneous assembly artifacts caused by low-quality nanopore reads, long-read contigs with an average short read coverage of 15x or less were discarded from the assembly. Assembly statistics and quality were recorded with QUAST (Gurevich et al., 2013) and CheckM (Parks et al., 2015), respectively. The sample was annotated with the NCBI Prokaryotic Genome Annotation Pipeline (PGAP) (Li et al., 2021; Tatusova et al., 2016).

Phylogenomic reconstruction was performed using the Up-to-date Bacterial Core Gene standalone software (UBCG) pipeline based on 92 conserved bacterial core genes (Na et al., 2018). All validly published *Labrys* type strains for which high-quality genome assemblies were publicly available in GenBank at the time of analysis were included, together with representative non-type genomes of the genus (Supplementary Table 2). The genome of *Labrys methylaminiphilus* JLW10^T^ was retrieved from the Joint Genome Institute (JGI) database. A complete list of all genomes included in the phylogenomic analysis, together with their strain designation and accession numbers, is provided in Supplementary Table 2. Core gene sequences were extracted, aligned, and concatenated using the UBCG pipeline, and a maximum-likelihood phylogenetic tree was inferred using IQ-TREE (Minh et al., 2020). The GTR evolutionary model was used to generate evolutionary distance matrices, and the robustness of the trees was assessed by 1,000 bootstrap replications. The tree was rooted using *Agrobacterium radiobacter* LMG 140^T^ as an outgroup, based on its established phylogenetic position within the *Rhizobiaceae*. The *in silico* DNA-DNA hybridization value and the G + C content percentage were obtained using the Genome-to-Genome Distance Calculator (GGDC) (Meier-Kolthoff and Göker, 2019), while the Average Nucleotide Identity (ANI) was calculated with OrthoANI v1.2 (Lee et al., 2016; Yoon et al., 2017b). Genes related to non-ribosomal peptide synthesis were determined by a search using the antiSMASH program (Blin et al., 2025).

### 2.4 Ecological distribution

To infer the broader ecological distribution of strain La1, its 16S rRNA gene and whole-genome sequence were analyzed using Protologger v2, a genome-informed pipeline that integrates taxonomic, genomic, and ecological metadata (Hitch et al., 2021). The platform assesses the presence and relative frequency of closely related 16S rRNA sequences across standardized amplicon datasets, identifying the ecological contexts in which *Labrys* La1-like sequences are most prevalent. Additionally, the genome was compared with publicly available metagenomics datasets to identify shared sequences with metagenome-assembled genomes (MAGs) generated from high-throughput microbial sequencing efforts.

### 2.5 Biochemical and chemotaxonomic analysis

Oxidase and catalase activities were assessed by adding oxidase reagent (bioMérieux) and 3% (v/v) hydrogen peroxide, respectively. Metabolic and physiological characteristics along with enzymatic activities were determined using API 20NE, API ZYM, and API 50CH commercial kits (bioMérieux) according to the manufacturer’s guidelines. Briefly, bacterial suspensions were prepared in sterile saline solution to the recommended turbidity, inoculated into test strips, and incubated at 28°C. Enzymatic activities and substrate utilization were scored after 24–48 h based on color development relative to negative controls, and reactions were classified as positive, weakly positive, or negative according to the intensity of the color change.

Cellular fatty acid profiles of strain La1 were determined after cultured in TSB (Tryptone Soy Broth) medium for 16 h at 28°C. Membrane fatty acids were extracted as described by Carrasco et al. (2023). Gas chromatography–mass spectrometry (GC–MS) analysis was carried out on a GC-MS QP2020NX (Shimadzu, Kyoto, Japan), equipped with an Rtx-5MS capillary column (Restek, 30 m × 0.25 mm i.d., 0.25 μm film thickness), and a single quadrupole mass spectrometer operating under electron ionization conditions (EI, 70 eV). Data were acquired in SCAN/SIM mode, and fatty acids were identified by comparing mass fragmentation patterns against the NIST 2008 mass spectral library. Fatty acid composition data for related members of the genus *Labrys*, including the type strain *Labrys methylaminiphilus* JLW10^T^, were retrieved from published sources and are included for qualitative contextual comparison only, as these datasets were generated under different cultivation and analytical conditions.

### 2.6 Plant growth-promoting and stress-protection assays

#### 2.6.1 Indole-3-acetic acid production

IAA production was assessed using the colorimetric Salkowski assay (Park et al., 2021). Strain La1 was cultured in LB supplemented with 0.1; 0.5; 1.0; 2.5; and 5.0 gL^–1^ L-tryptophan and incubated at 28°C for 48 h with shaking (150 rpm). Cultures were centrifuged at 8,000 × g for 5 min, and 100 μL of the supernatant was mixed with 200 μL of Salkowski reagent. After 30 min in darkness at room temperature, absorbance was measured at 536 nm, and IAA concentration was estimated using a standard curve prepared with IAA dissolved in methanol.

#### 2.6.2 Phosphate solubilization assays

The strain La1 was tested for phosphate solubilization from organic (phytase-screening medium (PSM), 10 gL^–1^ D-glucose, 4 gL^–1^ sodium phytate, 4 gL^–1^ CaCl_2_, 5 gL^–1^ NH_4_NO_3_, 0.5 gL^–1^ KCl, 0.5 gL^–1^ MgSO_4_ × 7H2O, 0.01 gL^–1^ FeSO_4_ × 7H_2_O, 0.01 gL^–1^ MnSO_4_ × H_2_O and 15 gL^–1^ agar) (Jorquera et al., 2018) and inorganic (Pikovskaya agar (PVK), 10 gL^–1^ D-glucose, 0.5 gL^–1^ yeast extract, 0.5 gL^–1^ (NH_4_)_2_SO_4,_0.1 gL^–1^ MgSO_4_.7H_2_0, 5 gL^–1^ Ca_3_(PO_4_)_2,_ 0.2 gL^–1^ KCl, 0.002 gL^–1^ MnSO_4_.2H_2_0, 0.002 gL^–1^ FeSO_4_.7H_2_0 and 15 gL^–1^ agar) (Nautiyal, 1999) phosphorus sources. The capacity to utilize phosphate was examined after incubation for 3–4 days at 28°C. The formation of clear halos around colonies was recorded as positive phosphate solubilization activity.

#### 2.6.3 Plant inoculation under osmotic and salt stress

To evaluate the protective effect of strain La1 on *L. luteus* seedlings, surface-sterilized seeds were germinated for 48 h on moist sterile filter paper. Uniform seedlings were transferred to Petri plates containing Murashige & Skoog medium (MS) (Murashige and Skoog, 1962) solidified with 1% agar, supplemented with either 2% (w/v) polyethylene glycol (PEG 6000) to simulate osmotic stress or 150 mM NaCl to simulate salt stress. Treatments included: (i) MS only (to determine La1 potential plant growth-promoting effect under non-stress conditions), (ii) osmotic stress (MS + PEG), and (iii) salt stress (MS + NaCl), inoculated and uninoculated with La1 strain (OD_600_ 0.6, 10^8^ CFU mL^–1^). Prior to inoculation, bacterial cells were harvested by centrifugation and washed twice with sterile buffer to minimize the carryover of external carbon sources. Plates were incubated at 22°C with a 16 h light/8 h dark cycle for 14 days. At the end of the experiment, plants were harvested, and their fresh weight was immediately recorded. Samples were then dried at 65°C for 48 h to determine dry weight.

#### 2.6.4 Antifungal activity assay

Antifungal activity of strain La1 was evaluated against *Colletotrichum lupini* and *Pleiochaeta setosa* using dual-culture antagonism assays as described by Campos et al. (2024), with minor modifications. Mycelial plugs (6 mm) were placed on PDA plates opposite a central streak of La1 culture. Plates were incubated at 22°C for 7 days, and fungal growth was quantified by measuring the radial distance (mm) from the fungal inoculation point to the edge of the mycelial front. Measurements were performed in six independent biological replicates per treatment.

### 2.7 Statistical analysis

Statistical analyses were performed using GraphPad Prism version 8.0 and R version 4.3.3. Prior to analysis, data normality was assessed using the Shapiro–Wilk test. For plant growth-promotion assays, paired *t*-tests were applied to compare inoculated and non-inoculated plants within each experimental condition, reflecting the paired experimental design. For fungal inhibition differences between control and La1 treatments were also evaluated using paired *t*-test Each treatment consisted of six independent biological replicates (*n* = 6). Results are reported as mean ± standard error (SE), and differences were considered statistically significant at *p* < 0.05.

## 3 Results

### 3.1 Morphological and physiological properties of strain La1

Under optimal growth conditions, colonies of strain La1 on YMB agar plates exhibited a beige appearance, smooth texture, and slightly mucoid consistency, with entire margins (Figure 1B). After 48 h of incubation at 28°C, colonies reached a diameter of approximately 1.0–1.5 mm. Microscopically, cells of strain La1 appeared as Gram-negative rods (Figure 1C). Growth occurred over a pH range of 5.0–8.0 and in the presence of up to 3% (w/v) NaCl. Optimal growth was observed at pH 6.0–7.0 and in media containing up to 1% (w/v) NaCl.

### 3.2 Genomic affiliation and comparative analysis

**FIGURE 2:**
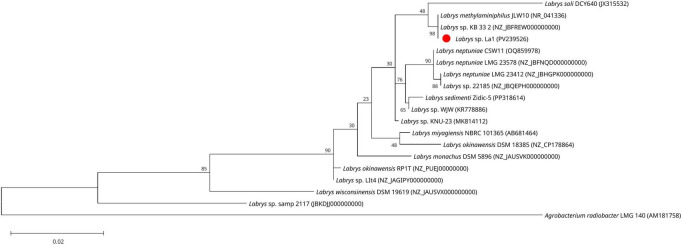
Phylogenetic analysis of strain La1 within the genus *Labrys*. Maximum-likelihood phylogenetic tree based on 16S rRNA gene sequences showing the placement of *Labrys* strain La1 (red dot) and its closest phylogenetic relatives. The tree was constructed using the TN93 substitution model. Bootstrap support values were calculated from 1,000 replicates. *Agrobacterium radiobacter* LMG 140^T^ was included as the outgroup.

The 16S rRNA gene sequence (1,490 nt) of strain La1 (GenBank accession PV239526) exhibited 99.2% similarity to *L. methylaminiphilus* JLW10^T^, followed by *L. miyagiensis* G24103 (98.5%), *L. neptuniae* Liujia-146 (98.3%). In phylogenetic analyses inferred by ML, strain La1 clustered closely with *L. methylaminiphilus* JLW10^T^, a strain isolated from lake sediment with methylotrophic activity (Miller et al., 2005), and the unclassified strain *Labrys* sp. KB 33_2 isolated from *Arabidopsis thaliana* (JBFREW010000000), and *Labrys soli* DCY64 isolated from rhizosphere of ginseng (Nguyen et al., 2015; Figure 2). This topology was consistent with that obtained using the NJ method, showing comparable branching patterns and further supporting the assignment of strain La1 to the genus *Labrys* (Supplementary Figure 1).

The hybrid ONT–Illumina assembly of strain La1 yielded a highly contiguous genome consisting of two circular contigs, with a total length of 7,351,893 bp and a GC content of 63.06%. The contigs measured 5,489,467 and 1,862,280 bp, with a sequencing coverage of 131x and 126x, respectively. The assembly yielded an N50 of 5,489,467 bp and an L50 of 1, indicative of a near-complete chromosome. No ambiguous bases (Ns) were detected. Genome quality assessment using CheckM, based on the Rhizobiales marker set, estimated 99.27% completeness and 1.99% contamination, confirming the high quality and suitability of the genome for taxonomic and comparative genomic analyses. Genome annotation revealed that the assembly contains 6,845 genes, including 6,691 coding sequences, 9 rRNA genes (3 copies for 5s, 16s, and 23s, respectively), 56 tRNA genes and 14 non-coding RNAs (ncRNAs).

Whole-genome comparison with publicly available *Labrys* genomes confirmed that La1 strain belongs to *L. methylaminiphilus*, showing ANI and dDDH values of 98.9 and 91.2%, respectively, with the type strain JLW10^T^ (Table 1). Accordingly, strain La1 was assigned to this species. Despite this high genomic relatedness, comparative genome annotation revealed the presence of gene clusters potentially associated with adaptation to acidic environments and plant-associated lifestyles, including stress-response systems, carbohydrate metabolism pathways (e.g., N-acetylglucosamine and β-glucosidase utilization), and exopolysaccharide biosynthesis, distinguishing La1 from previously described aquatic or soil-derived isolates.

**Table 1:** Comparative genomic similarity by ANI (green) and dDDH (blue) between strain La1 and *Labrys* genomes available in GenBank.

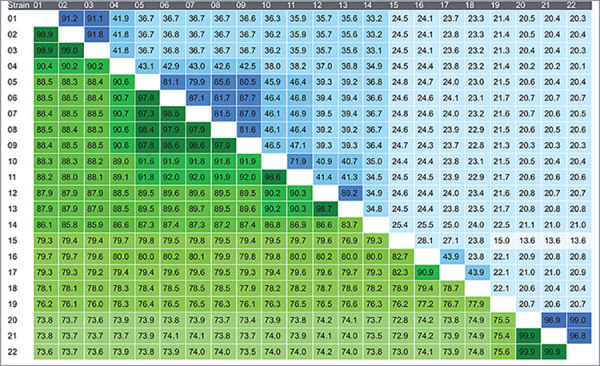

01, *Labrys* sp. La1; 02, *L. methylaminiphilus* JLW10^T^; 03, *Labrys* sp. KB 33_2; 04, *Labrys* sp. KNU-23; 05, *Labrys* sp. 22185; 06, *L. neptuniae* LMG 23578^T^; 07, *L. neptuniae* CSW11; 08, *L. neptuniae* LMG 23412; 09, *Labrys* sp. 1H043; 10, *L. sedimenti* Zidic-5^T^; 11, *Labrys* sp. WJW; 12, *Labrys* sp. LIt4; 13, *L. okinawensis* RP1T^T^; 14, *Labrys* sp. CB10b 03; 15, *Labrys* sp. ME2001; 16, *L. miyagiensis* NBRC 101365^T^; 17, *L. okinawensis* DSM 18385^T^; 18, *L. monachus* DSM 5896^T^; 19, *L. wisconsinensis* DSM 19619^T^; 20, *Labrys* sp. samp 2117 semibin 122; 21, *Labrys* sp. samp 2115 metabat2 227; 22, *Labrys* sp. samp 2119 metabat2 259.

**FIGURE 3:**
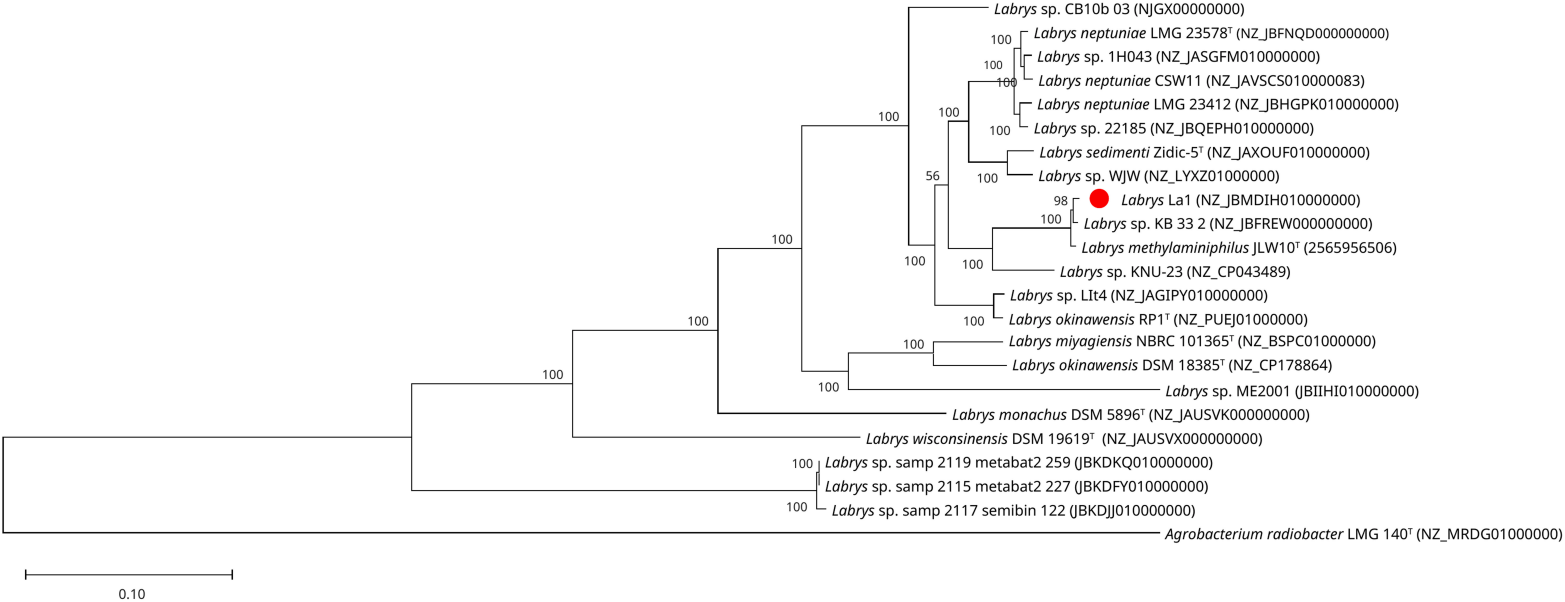
Phylogenomic analysis of strain La1 within the genus *Labrys*. Maximum-likelihood phylogenetic tree was constructed based on a concatenated alignment of 92 core genes using the UBCG pipeline. The analysis includes all the type strain genomes sequences published to date and the *Labrys* genomes available in GenBank database at the time of analysis, with the corresponding number access. Strain La1 is highlighted with a red dot. Type strains are indicated by a superscript “T.” *A. radiobacter* LMG 140^T^ was used as the outgroup. Bootstrap support values were calculated from 1,000 replicates.

The UBCG-based phylogenomic analysis, using both ML and NJ methods, revealed a well-supported topology for the genus *Labrys*. This analysis enabled the comparison of strain La1 with all available *Labrys* genomes deposited in the GenBank database, and *L. methylaminiphilus* JLW10^T^, using *A. radiobacter* LMG 140^T^ as an outgroup. The resulting phylogeny was congruent with the 16S rRNA gene-based analysis, with strain La1 clustering within the *L. methylaminiphilus* clade, closely associated with *Labrys* sp. KB 33_2 and forming a distinct but closely related lineage to the type strain *L. methylaminiphilus* JLW10^T^ (Figure 3 and Supplementary Figure 2). This clade grouped with *L. neptuniae* and *L. sedimenti*, in agreement with previously published phylogenetic reconstructions of the genus *Labrys* (Chou et al., 2007; Nguyen et al., 2015; Csépányi et al., 2025).

In addition to resolving the phylogenetic placement of strain La1, comparative genomic analyses revealed taxonomic inconsistencies among strains currently classified as *Labrys okinawensis*. Two clearly separated genomic groups were identified. The first group comprises strain DSM 18385^T^ and *L. miyagiensis* NBRC 101,365^T^, both originally isolated in Japan and previously characterized using phylogenetic, biochemical, and chemotaxonomic approaches (Islam et al., 2007). The second group includes strain *L. okinawensis* LIt4 (Chávez-Ramírez et al., 2022), isolated from root nodules of wild *Acaciella* sp. in Mexico, and the genome deposited as *L. okinawensis* RP1, a strain not formally published but listed in GenBank under this name. Phylogenomic reconstructions, together with low ANI (79.6%) and dDDH (24.0%) values between strains DSM 18385^T^ and LIt4, clearly separated these groups into distinct clades. These results suggest that LIt4, together with RP1, represent a species distinct from *Labrys okinawensis* and should therefore be designated as *Labrys* sp. until their formal taxonomic status is resolved.

### 3.3 Ecological association of *Labrys* La1

Phylogenetic analysis, ANI, and dDDH comparisons of the *Labrys* sp. La1 genome with available *Labrys* genomes, revealed its closest relationship to *L. methylaminiphilus* JLW10^T^, the type strain of the species isolated initially from lake sediment (Miller et al., 2005), and to *Labrys* sp. KB 33_2, which was recovered from the rhizosphere of *A. thaliana* Col-0 (Figure 3 and Table 1).

The ecological distribution of strain La1 was assessed using Protologger v2, based on both its 16S rRNA gene sequence and whole-genome data. Comparative analysis against publicly available metagenomic datasets did not identify any closely related metagenome-assembled genomes (MAGs), suggesting that genomes from this lineage are currently underrepresented in large-scale metagenomic reconstructions.

**FIGURE 4:**
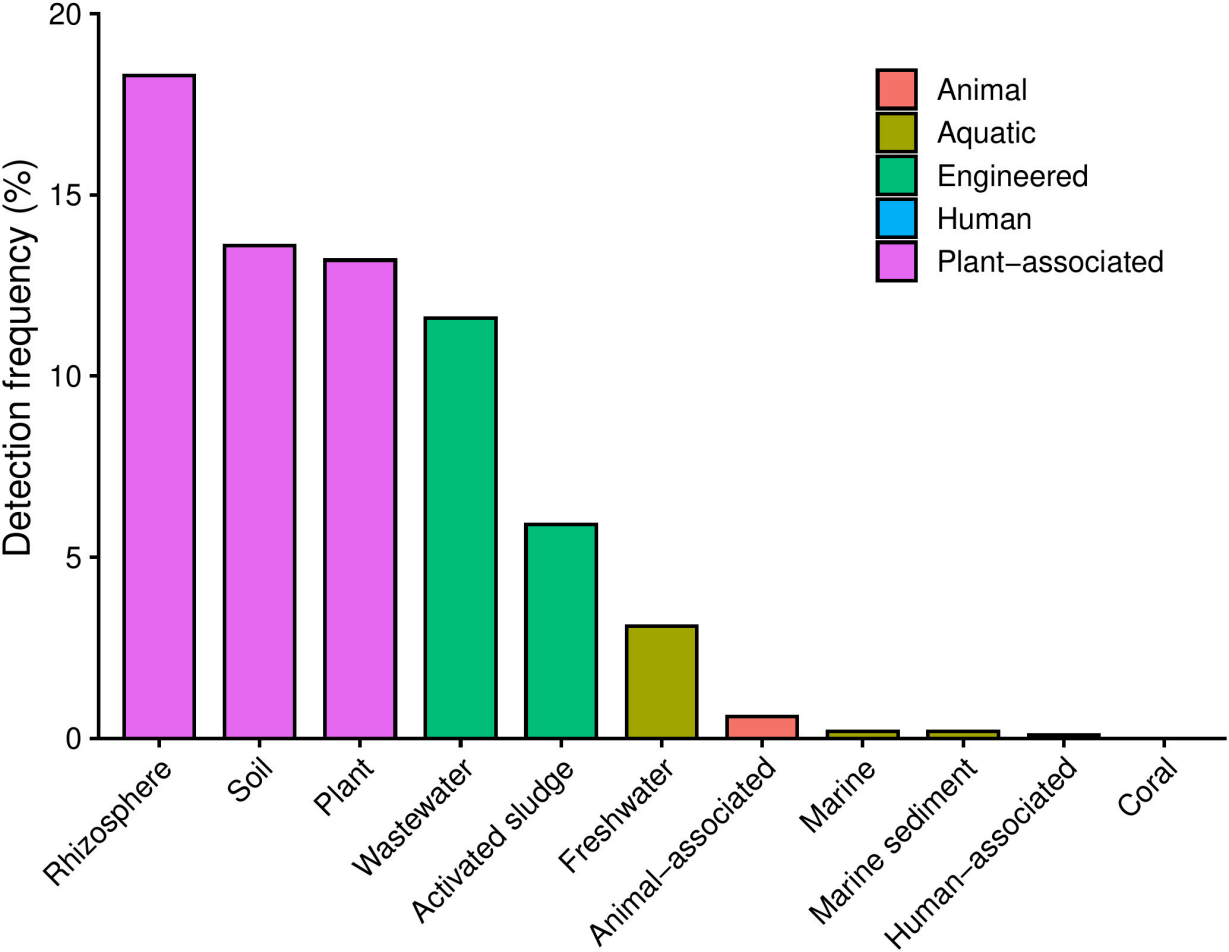
Environmental distribution of sequences related to *Labrys* sp. La1 in publicly available metagenomes. Bar plot showing the number of IMNGS-derived 16S rRNA sequence hits matching *Labrys* sp. La1 across different environmental metagenome categories. The highest number of hits was detected in soil, rhizosphere, and plant-associated metagenomes, followed by wastewater and gut microbiome datasets. In contrast, freshwater, aquatic, insect-associated, and dust metagenomes showed very low or no detectable matches.

Amplicon-based surveys revealed that sequences closely related to strain La1 are predominantly associated with terrestrial ecosystems. The highest detection frequencies were observed in rhizosphere microbiomes (18.3% of samples), plant-associated metagenomes (13.2%), and soil microbiomes (13.6%). Lower frequencies were found in wastewater (11.6%) and activated sludge (5.9%), whereas freshwater environments showed limited detection (3.1%) (Figure 4). In contrast, La1-like sequences were rarely detected in animal- or marine-associated microbiomes (including human, livestock, poultry, and marine environments) where detection frequencies remained consistently below 1%. Across all environments, mean relative abundances of La1-like sequences were low, consistent with a broad but low-abundance ecological distribution (Supplementary Table 1). These findings are derived from 16S rRNA gene amplicon datasets. They should be interpreted as relative detection patterns rather than quantitative measures of absolute abundance, given the inherent limitations of amplicon-based approaches. Consistent with these patterns, and providing independent support beyond amplicon-based surveys, phylogenetic analysis of 16S rRNA gene sequences showed that strain La1 clusters with several *Labrys* isolates recovered from *L. luteus* nodules collected in the same geographic region. These nodule-associated strains formed a coherent lineage within the *Labrys* genus, supporting a recurrent association of this clade with plant-root environments (Supplementary Figure 4).

### 3.4 Biochemical and chemotaxonomic characteristics

In biochemical profiling using API ZYM, API 20NE, and API 50CH systems, strain La1 exhibited enzymatic and metabolic traits consistent with members of the genus *Labrys*, while also showing distinctive features. Reactions were classified as positive when a clear and reproducible color change was observed, weakly positive when a faint but consistent reaction was detected, and negative when no color change occurred, following the interpretation criteria provided by the manufacturer. Strain La1 was positive for alkaline phosphatase, esterase (C4), leucine arylamidase, valine arylamidase, acid phosphatase, β-glucosidase, N-acetyl-β-glucosaminidase, and naphthol-AS-BI-phosphohydrolase, and weakly positive for oxidase activity. It assimilated D-glucose, D-mannose, D-mannitol, potassium gluconate, malate, and N-acetyl-D-glucosamine, but not maltose, sucrose, or citrate.

In the API 50CH system, acid production was observed from D-glucose, D-mannose, D-mannitol, and glycerol, whereas no acid was produced from sucrose, lactose, or maltose. The presence of both β-glucosidase and N-acetyl-β-glucosaminidase activities, together with the ability to utilize N-acetyl-D-glucosamine and potassium gluconate, distinguishes strain La1 from *L. methylaminiphilus* JLW10^T^ and other reference species of the genus. These characteristics suggest an enhanced capacity for the utilization of complex carbohydrates and osmolytes, which may contribute to its ecological adaptation to the rhizosphere environment of *Lupinus luteus*. The detailed biochemical properties of strain La1 and related species are summarized in Table 2.

**TABLE 2:** Biochemical characteristics of *L. methylaminiphilus* strain La1 and related species of the genus *Labrys*.

Test	1	2	3	4	5
**API 20NE/API ZYM**					
Arginine dihydrolase^*^	-	+	n	n	n
Urease	-	-	n	n	n
Oxidase	w	+	+	+	-
Catalase^*^	-	+	n	+	n
β-Galactosidase	-	-	-	-	-
Nitrate reduction	w	+	n	n	n
Indole production	-	-	n	n	n
Aesculin hydrolysis^*^	+	-	n	n	n
Gelatin hydrolysis^*^	-	w	n	n	n
**Assimilation of carbon sources**					
Malate	+	+	n	n	n
Sodium citrate^*^	-	+	n	n	n
Potassium gluconate	+	n	+	+	+
Glucose	+	+	+	n	n
Maltose^*^	-	+	n	n	n
Mannose	+	+	+	n	n
Sucrose^*^	-	+	n	n	n
Mannitol	+	+	+	n	n
**API 50CH—Acid production from carbohydrates**					
Glycerol^*^	-	+	+	+	+
Arbutin	+	n	-	-	+
Sucrose	-	-	-	-	+
D-Ribose	-	n	+	+	+
D-Galactose	-	n	+	+	+
D-Glucose	+	+	+	+	+
D-Fructose^*^	-	+	+	+	+
D-Mannose	+	+	+	+	+
D-Mannitol	+	+	+	+	+
D-Maltose^*^	-	+	-	-	+
D-Lactose	-	-	-	-	+
N-Acetyl-D-glucosamine	+	n	-	-	-
Potassium-2-ketogluconate	+	n	-	-	-

1, La1 (This study)^1^; 2, *L. methylaminiphilus* JLW10*^T^* (Miller et al., 2005; Albert et al., 2010; Carvalho et al., 2008); 3, *Labris sedimenti* Zidic-5*^T^* (Csépányi et al., 2025); 4, *Labrys portucalensis* LMG 23412*^T^* (Csépányi et al., 2025); 5, *Labrys neptuniae* LMG 23578^T^ (Csépányi et al., 2025). +, positive; -, negative; w, weak reaction; n, data not available. Traits marked with an asterisk indicate phenotypic differences between strain La1 and the type strain. Differences are reported based on assays performed under the conditions used in this study and reference data obtained from the literature.

The major cellular fatty acids (> 10%) identified in *Labrys* sp. strain La1 were C18:1 ω7c (31.0%), C14:0 3-OH (17.2%), C12:0 (13.6%), C18:0 (11.7%), and C16:0 (11.6%). This profile is consistent with those reported for other members of the genus *Labrys*, particularly the predominance of C18:1 ω7c as the principal unsaturated fatty acid (Carvalho et al., 2008).

Strain La1 exhibited relatively elevated proportions of saturated and hydroxylated fatty acids, a trait commonly linked to enhanced membrane stability under acidic or osmotic stress (Eberlein et al., 2018). Although the overall fatty acid profile shows qualitative similarities with related taxa, direct quantitative comparisons should be interpreted cautiously, as reference data, such as those for *L. methylaminiphilus* JLW10^T^, derived from studies employing different cultivation and analytical protocols (Miller et al., 2005).

Cyclopropane fatty acids (C19:0 cyclo ω7c and C19:0 cyclo ω8c), previously reported in some *Labrys* species, were not detected in strain La1 under the tested growth conditions. The complete fatty acid profiles of strain La1 and selected *Labrys* species are summarized in Table 3.

**TABLE 3:** Cellular percentage of fatty acid content of La1 strain and related species of the *Labrys* genus.

Fatty acid	1	2	3	4	5	6	7	8	9	10
C_12:0_	13.6	–	–	–	–	–		–	–	–
C_16:0_	11.6	17.7	16.5	17.7	15.5	13.6	12.4	9.7	12.9	16.2
C_17:0_	1.2	–		–		–	–	–	–	–
C_18:0_	11.7	1.7	4.0	2.0	2.9	2.0	2.2	5.2	3.5	2.1
C_14:0_ 3-OH	17.2	–	2.4	3.1	2.0	–		–	–	–
C_16:0_ 2-OH	–	–	–	–	–	-	TR	1.0	TR	-
C_16:0_ 3-OH	–	–	–	–	–	-	3.7	1.3	1.5	2.1
C_18:1_ 2-OH	–	–	–	–	–	-	TR	1.2	TR	-
C_18:0_ 3-OH	8.5	–	1.1	ND	TR	2.2	TR	3.6	3.1	3.6
C_16:1_ ω7c	1.2	–	–	–		–		–	–	–
C_18:1_ ω7c	31.0	32.8	60.0	45.0	47.7	–	–	–	–	–
C_18:0_ cyclo ω8c	2.5	–	–	–	–	–	–	–	–	–
C_19:0_ cyclo ω7c	–	49.4	13.0	27.8	28.4	–	–	–	–	–
C_19:0_ cyclo ω8c	–	–	–	–	–	16.8	38.2	29.1	34.6	16.1
C_18:1_ ω7c 11-methyl	–	–	1.4	1.1	TR	–		–	–	–
Summed features 2	–	–	–	–	–	4.0	4.0	4.3	4.1	5.4
Summed features 4	–	–	–	–	–	-	-	-	-	1.3
Summed features 8	–	–	–	–	–	61.4	36.0	43.2	38.0	52.4

1, strain La1^1^; 2, *L. methylaminiphilus* JLW10^T^ (Miller et al., 2005); 3, *L. sedimentis* Zidic-5^T^ (Csépányi et al., 2025); 4, *L. portucalensis* LMG 23412 (Csépányi et al., 2025)^2^; 5, *L. neptuniae* LMG 23578^T^ (Csépányi et al., 2025); 6, *L. soli* DCY64 (Nguyen et al., 2015); 7, *L. monachus* JCM 21795 (Nguyen et al., 2015); 8, *L. okinawensis* DSM 18385^T^ (Nguyen et al., 2015); 9, *L. miyagiensis* KCTC 22027 (Nguyen et al., 2015); 10, *L. portucalensis* DSM 17916 (Nguyen et al., 2015). Summed features represent groups of two or three fatty acids that could not be separated by GC with the MIDI system. Summed feature 2 comprises C12:0 aldehyde and/or unknown fatty acid of equivalent chain length 10.928. Summed feature 4 comprises C17:1 iso I and/or anteiso B. Summed feature 8 comprises C18:1 ω7c and/or C18:1 ω6c.

### 3.5 Functional genomic

Genome mining of strain La1 using antiSMASH identified biosynthetic gene clusters (BGCs) including those predicted to encode non-ribosomal peptide synthetases (NRPS), siderophore biosynthesis, and molecules involved in oxidative stress tolerance (Table 4). Comparative genomic analysis with strains JLW10^T^ and KB 33_2 revealed a broadly conserved BGC repertoire across all three genomes, consistent with their close phylogenomic relatedness (Figure 3 and Supplementary Figure 3). Among the detected clusters, gene sequences with similarity to BGCs encoding rhizomide A/B/C, and the siderophore ochrobactin were present in all three strains. These annotations are based on *in silico* predictions, and no experimental validation of compound production was performed. Accordingly, these findings reflect biosynthetic potential rather than confirmed metabolite expression or function. Beyond secondary metabolism, genome analysis also revealed genes related to acid resistance, phosphate uptake, and carbohydrate-active enzymes, supporting adaptation to the acidic environment. These features collectively support the ecological inference that strain La1 is adapted to acidic, oligotrophic soils characteristic of southern Chile.

**TABLE 4:** Classification of biosynthetic gene clusters (BGCs) identified by antiSMASH with predicted antifungal activity.

Strain	NRPS	PKS	RIPP	Siderophores	Terpenes
La1	1	1	4	1	2
JLW10^T^	1	1	4	1	2
KB 33_2	1	1	4	1	2

### 3.6 Plant growth-promoting and stress-protective traits of *L. methylaminiphilus* La1

**FIGURE 5:**
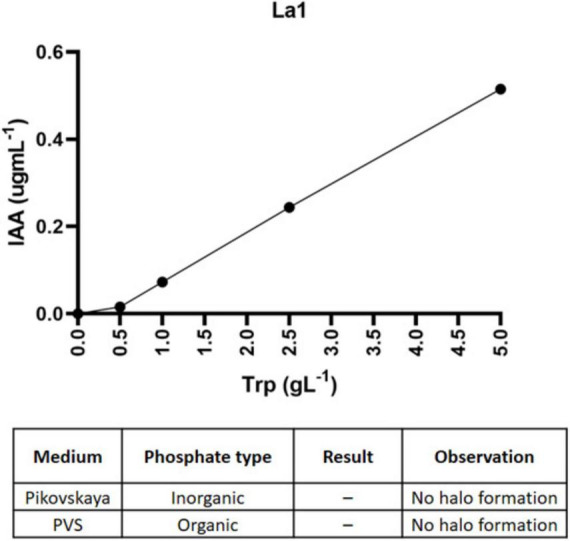
Evaluation of plant growth-promoting activities in *L. methylaminiphilus* La1. Line plot shows indole-3-acetic acid (IAA) production in response to increasing concentrations of L-tryptophan after 48 h of incubation in LB medium. The table below summarizes phosphate-solubilization results on Pikovskaya and PVS media.

Strain La1 tested positive for IAA production in the Salkowski assay after 48 h of incubation in LB medium supplemented with L-tryptophan. In contrast, no phosphate solubilization was detected on Pikovskaya agar (inorganic phosphate) or in PVS medium (organic phosphate), indicating that phosphorus mobilization is not a primary metabolic trait of this strain (Figure 5).

**FIGURE 6:**
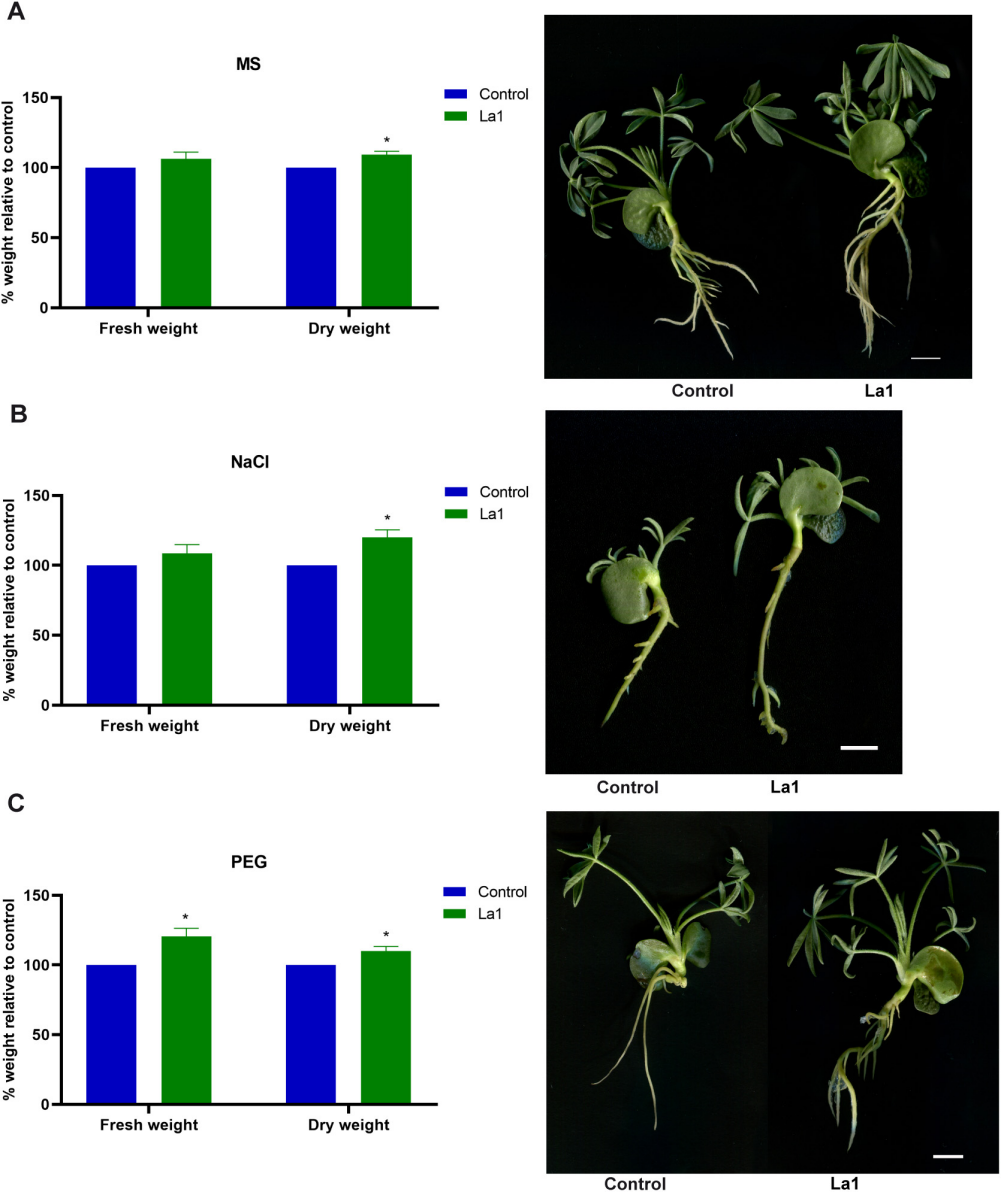
Effect of *L. methylaminiphilus* strain La1 inoculation on *L. luteus* seedlings under normal and stress conditions. **(A)** Plant growth-promoting activity (MS medium), **(B)** 150 mM NaCl, and **(C)** 2% PEG-6000. Seedlings were evaluated for fresh and dry weight after 15 days of growth. Bars represent mean ± standard deviation (*n* = 6). Asterisks (*) indicate significant differences compared with the uninoculated control according to Student’s *t*-test (*p* < 0.05). Representative plants from each treatment are shown on the right. Scale bars = 1 cm.

To further assess its potential benefits to the host plant under abiotic stress, *L. luteus* seedlings were subjected to osmotic (PEG 2%) and salt (NaCl 150 mM) stress, with or without inoculation with strain La1 (Figure 6). Inoculated plants consistently exhibited higher biomass accumulation than uninoculated controls. Under non-stress conditions (MS medium), La1 increased both fresh and dry weight by approximately 15–20% (Figure 6A). Under salinity (NaCl) (Figure 6B) and osmotic (PEG) stress (Figure 6C), the positive effect was more pronounced, with inoculated plants showing significant increases (*p* < 0.05) in fresh and dry biomass compared with controls. Visual inspection revealed that La1-inoculated seedlings displayed more developed root systems and greener shoots, suggesting a mitigation of stress effects and improved plant vigor after 7 days.

### 3.7 Antifungal activity against lupin pathogens

**FIGURE 7:**
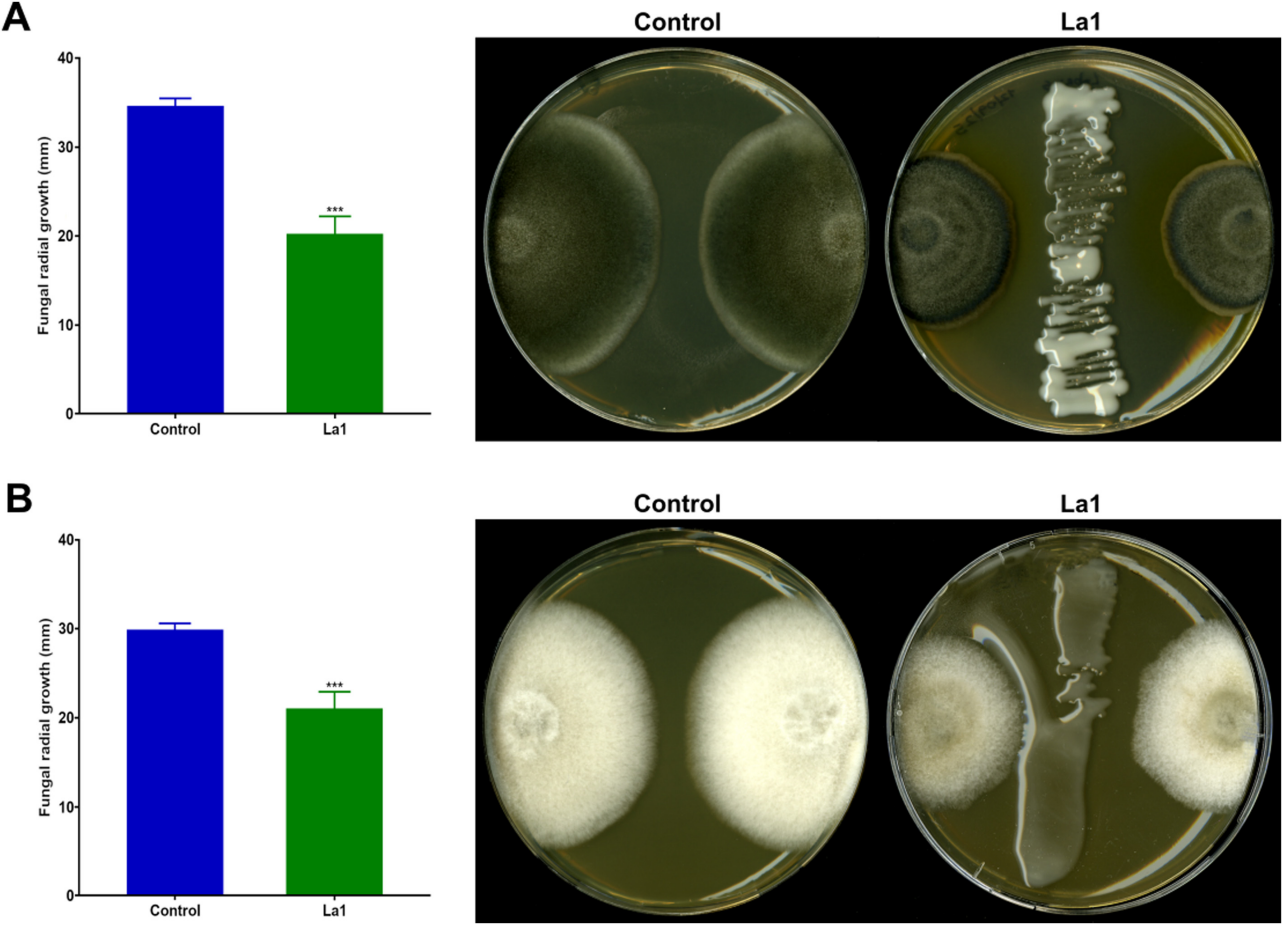
Antifungal activity *L. methylaminiphilus* strain La1 against phytopathogenic fungi. Dual culture assays on PDA plates were used to evaluate the antagonistic activity of strain La1 against phytopathogenic fungi. **(A)** *P. setosa* and **(B)** *C. lupini* were co-cultured with strain La1 for 7 days at 25°C. Representative images show fungal growth in control plates and in the presence of La1. Antifungal activity was evidenced by a significant reduction in radial mycelial growth toward the bacterial streak compared with uninoculated controls. Bar graphs show mean fungal radial growth ± standard deviation (*n* = 6). Asterisks (***) indicate statistically significant differences compared with the control according to Student’s *t*-test (*p* < 0.05).

The antagonistic activity of *L. methylaminiphilus* strain La1 was evaluated against two phytopathogenic fungi, *P. setosa* and *C. lupini*, using dual-culture assays on PDA plates. After 7 days of incubation at 25°C, strain La1 markedly inhibited the radial growth of both pathogens compared with the uninoculated controls (Figure 7). A restricted mycelial expansion toward the bacterial streak was observed, with a distinct inhibition zone separating bacterial and fungal growth fronts. These results indicate that La1 produces diffusible antifungal compounds capable of suppressing mycelial development of important lupine pathogens, suggesting a potential biocontrol role within the rhizosphere of *L. luteus*.

## 4 Discussion

The phenotypic, genomic, and ecological analyses presented here consistently support that *L. methylaminiphilus* strain La1 represents a rhizosphere-adapted lineage within the genus *Labrys*, exhibiting traits distinct from those previously described. Although members of the genus *Labrys* have been isolated from diverse environments, including aquatic sediments, soils, and plant-associated habitats (Miller et al., 2005; Chou et al., 2007; Islam et al., 2007; Nguyen et al., 2015; Chávez-Ramírez et al., 2022; Csépányi et al., 2025), their ecological roles within plant-associated microbiomes remain poorly characterized. By integrating strain-level phenotypic traits, genome-based analyses, and functional assays, this study provides new insights into the taxonomy, function, and ecological niche; as well as how members of *Labrys* may contribute to plant-associated microbial communities.

The morphological and physiological characteristics of strain La1 are consistent with those described for other members of the genus *Labrys*. Colonies were beige, convex, and slightly mucoid, and cells exhibited the typical Gram-negative rod morphology shared by all validly described *Labrys* species (Albert et al., 2010; Carvalho et al., 2008; Chou et al., 2007; Csépányi et al., 2025; Islam et al., 2007; Miller et al., 2005; Nguyen et al., 2015). The mucoid colony phenotype suggests the production of exopolysaccharides, a common trait among plant-associated bacteria that facilitates root adhesion, biofilm formation, and protection against environmental fluctuations (Carezzano et al., 2023). These characteristics are particularly relevant in the rhizosphere, where microorganisms experience dynamic changes in moisture, nutrient availability, and pH.

Strain La1 showed robust growth over a pH range of 5.0–8.0 and tolerated NaCl concentrations up to 3%, indicating a neutrophilic and moderately halotolerant physiology (Egamberdieva et al., 2019). These traits are consistent with adaptation to the acidic, nutrient-poor volcanic soils typical of southern Chile (Borie et al., 2019), where *L. luteus* is cultivated. The optimal growth range (pH 6–7) is compatible with rhizosphere conditions, supporting the ecological compatibility of La1 with the lupin root environment under field conditions.

The biochemical and enzymatic profiles of La1 further revealed traits suggestive of ecological specialization. The presence of β-glucosidase and N-acetyl-β-glucosaminidase activities, which are absent in the type strain *L. methylaminiphilus* JLW10^T^ (Miller et al., 2005), indicates an enhanced capacity to degrade complex carbohydrates and chitin-derived substrates. These enzymatic activities are commonly associated with saprophytic and plant-associated bacteria that utilize root exudates and decompose organic matter in the rhizosphere (Solheim and Fjellheim, 1984; Veliz et al., 2017). In addition, the ability of La1 to assimilate potassium gluconate, malate, and mannitol suggests metabolic flexibility and osmotic tolerance, traits that are advantageous under fluctuating soil conditions (Bais et al., 2006; Venturi and Keel, 2016). In contrast, the narrower carbon utilization of JLW10^T^, originally isolated from methylamine-rich aquatic sediments (Miller et al., 2005), reflects a distinct ecological specialization, highlighting a functional divergence within species.

From a genomic perspective, La1 shows high ANI (98.9%) and dDDH (91.2%) values similar to those of *L. methylaminiphilus* JLW10^T^, supporting its classification within this species. While not all phenotypic traits historically described for the species were reassessed under standardized conditions alongside the type strain, whole-genome-based metrics currently represent the most robust and widely accepted criteria for species assignment and subspecies-level differentiation in bacterial systematics.

However, phylogenomic analyses based on conserved core genes revealed a coherent lineage comprising La1 and other plant-associated isolates, distinct from aquatic or sediment-derived *Labrys* species. This ecological differentiation is reinforced by metagenomic surveys, which show that sequences closely related to La1 are predominantly detected in soil, rhizosphere, and plant-associated datasets rather than in aquatic environments. Together, these findings provide strong evidence that La1 occupies a terrestrial, plant-associated ecological niche.

It should be noted that inferences drawn from 16S rRNA gene amplicon datasets are subject to inherent biases, including uneven environmental representation, differences in sequencing depth, and limited resolution at the strain or subspecies level. Therefore, the ecological patterns reported here should be interpreted as indicative of preferential association rather than strict ecological specialization. Within this framework, the consistent detection of La1-related sequences across independent soil, rhizosphere, and plant-associated datasets provides convergent support for a plant-associated ecological niche.

An independent phylogenomic reconstruction using the EasyCGTree pipeline (Zhang et al., 2023) yielded a topology that was largely congruent with the UBCG-based analysis at the species level (Supplementary Figure 5). However, EasyCGTree showed branch contraction among closely related lineages within *L. methylaminiphilus*, resulting in reduced resolution at the subspecies level. This behavior is consistent with protein-based phylogenies, in which synonymous nucleotide variation on codons is not captured, and fine-scale evolutionary divergence may be underestimated. In this context, the nucleotide-based UBCG framework provides greater resolving power for closely related lineages and is therefore more appropriate for subspecies-level inference in this study.

Chemotaxonomic observations in strain La1 are broadly consistent with its proposed ecological niche but were not used as primary evidence in its classification. Under the tested growth conditions, La1 exhibited a fatty acid profile enriched in saturated (C12:0, C16:0, C18:0) and hydroxylated fatty acids (C14:0 3-OH, C18:0 3-OH), alongside a low abundance or complete absence of cyclopropane fatty acids. Such membrane compositions have been frequently associated with increased rigidity and enhanced tolerance to acidic and osmotic stress (Eberlein et al., 2018).

Given the strong influence of cultivation parameters on fatty acid profiles, these chemotaxonomic traits were interpreted with caution and framed primarily in a physiological rather than taxonomic context. Within this framework, the observed profile is compatible with survival in acidic, oligotrophic soils, where pH fluctuations, osmotic pressure, and nutrient scarcity impose selective constraints (Walczak-Skierska et al., 2024).

This physiological interpretation is further supported by genomic data, including the presence of acid-resistance mechanisms and carbohydrate-active enzyme repertoires commonly linked to rhizosphere-adapted lifestyles (Trivedi et al., 2020). Taken together, genomic, ecological, and physiological evidence supports the classification of strain La1 as a plant-associated *Labrys* lineage, with chemotaxonomic features serving as complementary, rather than diagnostic, indicators.

As previously described, *Labrys* sp. have been primarily described as free-living soil or aquatic bacteria. Several studies have reported the occurrence of *Labrys*-related strains in association with plant tissues, including root nodules of leguminous plants (Chou et al., 2007; Hossain and Lundquist, 2016; Gerding et al., 2017; Tapia-García et al., 2020; Chávez-Ramírez et al., 2022). Importantly, the presence of *Labrys* in nodules does not imply a canonical rhizobial symbiosis or a direct role in nitrogen fixation. Instead, these bacteria are more likely to inhabit nodules as non-rhizobial endophytes or nodule-associated microorganisms, a phenomenon increasingly recognized in both wild and cultivated legumes. Such non-rhizobial inhabitants may persist within nodules by exploiting available carbon sources or protected microenvironments and may contribute indirectly to plant performance through mechanisms such as stress tolerance, modulation of microbial interactions, or production of bioactive metabolites (Hnini and Aurag, 2024; Hassen et al., 2025). Consistent with this interpretation, the genome of strain La1 lacks key nodulation and nitrogen fixation genes, supporting a plant-associated but non-symbiotic lifestyle.

The ecological distribution patterns inferred from Protologger analyses provide a genome-informed perspective on the environmental contexts in which sequences closely related to strain La1 have been detected. Amplicon-based surveys revealed that La1-like 16S rRNA gene sequences are predominantly associated with terrestrial habitats, showing higher detection frequencies in rhizosphere, plant-associated, and soil microbiomes. At the same time, animal- and marine-related environments consistently exhibited low or negligible detection rates. Across all environmental categories, relative abundances were low, suggesting that this lineage is widespread but rare. Importantly, these amplicon-based observations are corroborated by independent, culture-based evidence from phylogenetic analyses of 16S rRNA gene sequences. In addition to strain La1, multiple *Labrys* isolates (B650 series) obtained from root nodules of *L. luteus* in the same geographic region clustered within the same lineage, forming a coherent clade within the genus. These isolates were recovered through targeted cultivation and are therefore not subject to the sampling biases inherent to large-scale amplicon datasets.

The repeated isolation of phylogenetically related *Labrys* strains from plant nodules provides direct empirical support for a consistent association of this lineage with plant-root environments at the local scale. In this context, *L. neptuniae* LMG 23578^T^ and *L. okinawensis* LIt4 were isolated from nodules of *Neptunia oleracea* and *Acaciella* sp., respectively (Chou et al., 2007; Chávez-Ramírez et al., 2022), supporting the occurrence of members of this genus within root nodules. In addition, a study conducted on Adesmia species in the south-central zone of Chile reported the presence of *Labrys* in root nodules of *Adesmia emarginata* (Gerding et al., 2017). Phylogenetic analysis based on 16S rRNA gene sequences revealed that the *Labrys*-associated isolates formed a clade with *L. methylaminiphilus*, supporting their relationship. When considered together, the convergence of amplicon-derived patterns and culture-based phylogenetic evidence supports the interpretation that strain La1 belongs to a plant-associated *Labrys* lineage.

As described in the previous section, comparative genomic analyses revealed taxonomic inconsistencies within strains assigned to *Labrys okinawensis*. Phylogenetic and phylogenomic reconstructions separated two distinct clades, supported by low ANI and dDDH values, indicating that some strains currently grouped under this name require taxonomic re-evaluation. A similar nomenclatural adjustment was previously made for strain LMG 2341, initially identified as *L. portucalensis* (Carvalho et al., 2008) and later reclassified as *Labrys neptuniae* based on genomic evidence (Csépányi et al., 2025). Although not central to the present study, these results highlight the value of genome-based approaches for resolving ecological and evolutionary relationships within the genus *Labrys*.

Genome mining of strain La1 revealed the presence of biosynthetic gene clusters associated with siderophore biosynthesis, non-ribosomal peptide synthetases (NRPS), oxidative stress tolerance, and carbohydrate-active enzymes, indicating genomic potential for secondary metabolite production and adaptation to competitive soil environments. The predicted capacity to synthesize siderophores and NRPS-derived metabolites suggests a potential role in micronutrient acquisition, rhizosphere competition, and metal scavenging, traits commonly associated with rhizosphere-adapted bacteria (Mendes et al., 2013; Scavino and Pedraza, 2013). In this context, the antifungal activity observed *in vitro* against *P. setosa* and *C. lupini* is consistent with the predicted biosynthetic potential of strain La1. However, as genome mining does not confirm metabolite expression or biological activity, the specific compounds underlying the antagonistic phenotype were not identified in this study. Therefore, the contribution of individual biosynthetic gene clusters to the observed antifungal effects remains putative and warrants further investigation using targeted metabolomic, gene expression, or functional approaches. Collectively, these genomic and phenotypic observations support the interpretation of strain La1 as a rhizosphere-adapted bacterium with the potential to modulate microbial interactions, while emphasizing the need for future functional validation.

Functionally, strain La1 produced IAA in a tryptophan-dependent manner, a phytohormone known to stimulate root development, enhance nutrient foraging, and modulate plant stress responses (Etesami and Glick, 2024). Although phosphate solubilization was not detected, inoculation assays demonstrated that La1 significantly increased fresh and dry biomass of *L. luteus* seedlings under both control and stress conditions. Notably, the growth-promoting effect was more pronounced under salinity and osmotic stress, suggesting that La1 may mitigate stress-related growth inhibition through hormone-mediated effects, osmoprotection, or improved root system architecture.

Importantly, the functional traits observed in strain La1 do not imply obligate symbiosis or direct biocontrol activity. Instead, they indicate that La1 may contribute to plant performance and stress resilience within a broader microbial community, consistent with current views of rhizosphere function as an emergent property of complex microbial assemblages.

In summary, the combined phenotypic, chemotaxonomic, genomic, and functional evidence supports the interpretation of L. *methylaminiphilus* strain La1 as a rhizosphere-adapted lineage with distinctive ecological capabilities. In this context, the subspecies designation is proposed to reflect stable, genome-supported ecological differentiation rather than transient phenotypic plasticity or strain-level variation. Its tolerance to acidic soils, production of IAA, genomic potential for secondary metabolite synthesis, and antagonistic activity against phytopathogenic fungi highlight the ecological versatility of the genus *Labrys* and its relevance within plant-associated microbiomes. These findings provide a robust ecological framework supporting the proposal of *L. methylaminiphilus* subsp. *lupini* subsp. nov., while emphasizing the importance of strain-level analyses for understanding microbiome-mediated plant resilience in challenging soil environments.

## 5 Conclusion

In this study, we characterized *Labrys methylaminiphilus* strain La1, isolated from *L. luteus* nodules in acidic soils of southern Chile. We identified it as a distinct plant-associated lineage within the species. Although La1 shares high genomic similarity with *L. methylaminiphilus* JLW10^T^, its physiological, biochemical, chemotaxonomic, and ecological traits collectively differentiate it from previously described aquatic and soil-derived isolates. In particular, La1 tolerates acidic and moderately saline conditions, metabolizes carbohydrates relevant to the rhizosphere, and displays a fatty acid profile consistent with adaptation to terrestrial environments subject to environmental stress.

Genomic analyses revealed the presence of genes associated with plant interaction, stress tolerance, siderophore biosynthesis, and NRPS pathways, indicating genomic potential for secondary metabolite production and a rhizosphere-adapted lifestyle. These genomic features are consistent with laboratory assays demonstrating IAA production, enhanced plant biomass under salinity and osmotic stress, and antagonistic activity against major lupin fungal pathogens. In addition, IMNGS-based metagenomic surveys showed that sequences closely related to strain La1 are predominantly detected in soil, rhizosphere, and plant-associated microbiomes, further reinforcing its ecological association with plant-root environments.

These results indicate that strain La1 represents a functionally versatile and ecologically specialized lineage of *L. methylaminiphilus* with potential relevance for plant performance in acidic soils. Based on the integration of physiological, chemotaxonomic, genomic, and ecological evidence, we propose the designation *Labrys methylaminiphilus* subsp. *lupini* subsp. nov. This subspecies expands the known ecological range of the genus *Labrys* and highlights its previously underappreciated association with legume-associated microbiomes. Future studies should aim to validate the ecological functions inferred here under field conditions, to identify the metabolites underlying the observed antagonistic activity, and to elucidate the molecular mechanisms by which strain La1 contributes to plant performance within complex microbial communities across diverse soil environments.

## Data Availability

The whole-genome shotgun (WGS) project for *Labrys methylaminiphilus* strain La1 has been deposited in GenBank under accession number JBMDIH000000000, BioProject PRJNA1233246, and BioSample SAMN47264006. The genome sequence is publicly available. Strain La1 has also been deposited in two publicly accessible international culture collections: the Chilean Collection of Microbial Genetic Resources (CChRGM) under accession RGM 3740 and the BCCM/LMG Bacteria Collection under accession LMG 34088. All other relevant data supporting the findings of this study are included within the article and its Supplementary Material. Additional information is available from the corresponding author upon reasonable request.
