# Supplementary material for: Functional and ecological characterization of Labrys methylaminiphilus subsp. lupini subsp. nov., associated with Lupinus luteus nodules in acidic soils of southern Chile
Source: Front Microbiol. 2026 Mar 9;17:1759558. doi: 10.3389/fmicb.2026.1759558 (PMC13006612; doi:10.3389/fmicb.2026.1759558)
Supplement: Supplementary file 1 [file Data_Sheet_1.pdf]

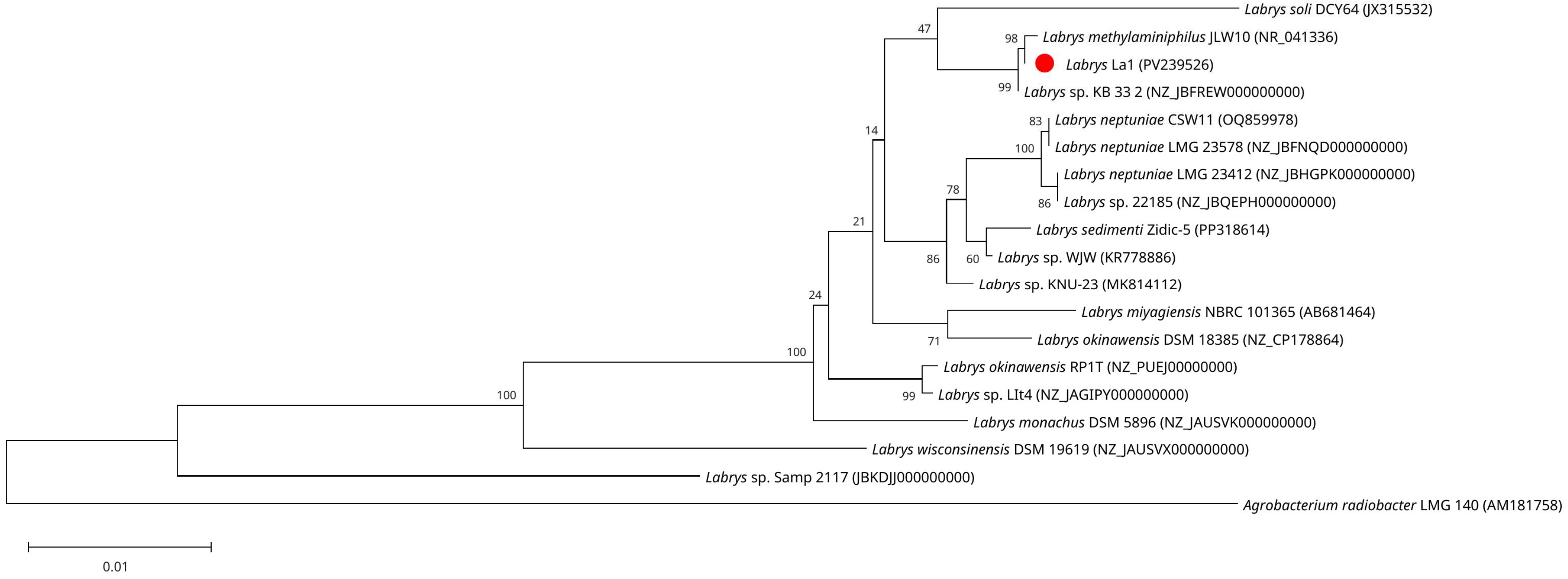

Figure S1. Phylogenetic analysis comparing strain La1 with different strains of the genus *Labrys*. Neighbor-joining phylogenetic tree based on 16S rRNA gene sequences, showing the position of *Labrys* strain La1 (red spot) and its closest phylogenetic relatives. The tree was constructed using the HKY85 substitution model. Bootstrap values were calculated from 1000 replicates to assess branch support. *Agrobacterium radiobacter* LMG 140^T was included as the outgroup.

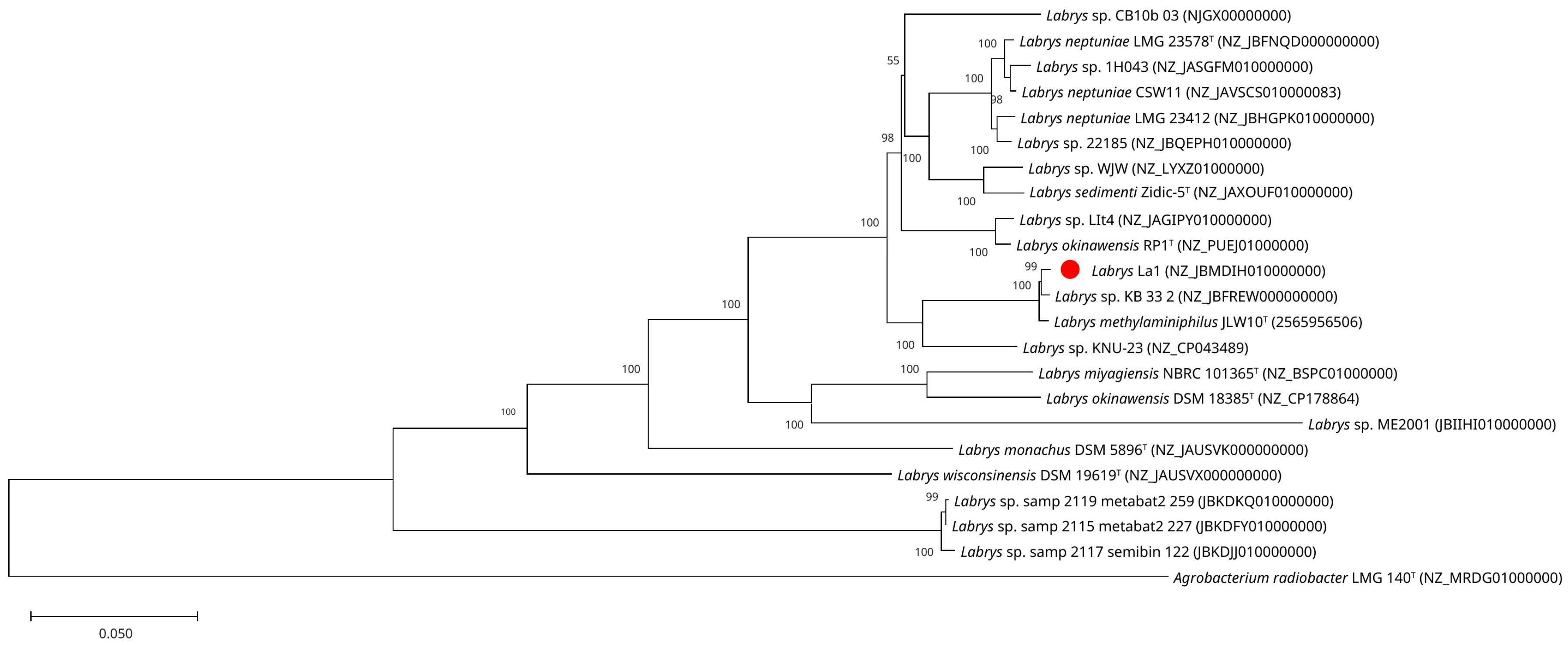

Figure S2. Phylogenetic analysis comparing strain La1 genome with the genomes strains of the genus *Labrys* accessible from GenBank database. Neighbor-joining phylogenetic tree based on concatenated alignment of 92 core genes, showing the position of *Labrys* strain La1 (red spot). Bootstrap values were calculated from 1000 replicates to assess branch support. *A. radiobacter* LMG 140^{T} was included as the outgroup.

A

Region	Type	From	To	Similarity Confidence	Most similar known cluster	
Region 1.1	NRPS, T1PKS, hserlactone	1,289,829	1,337,889	High	rhizomide A/rhizomide B/rhizomide C	NRPS:Type I
Region 1.2	terpene	1,699,137	1,720,024			
Region 1.3	RRE-containing	1,848,532	1,868,825			
Region 1.4	NAPAA	4,273,293	4,307,354			
Region 1.5	terpene-precursor, RiPP-like	4,772,790	4,803,801	Low	polyhydroxyalkanoate	other:other
Region 1.6	thioamitides	5,111,671	5,138,171			
Region 2.1	NI-siderophore	47,105	79,839	High	ochrobactin	other:other
Region 2.2	redox-cofactor	317,013	339,158			
Region 2.3	RiPP-like	624,172	634,978			
Region 2.4	hserlactone	1,228,636	1,249,286			
Region 2.5	RiPP-like	1,457,245	1,468,051			

B

Region	Type	From	To	Similarity Confidence	Most similar known cluster	
Region 2.1	hserlactone	358,094	378,744			
Region 3.1	NAPAA	268,479	302,540			
Region 5.1	NI-siderophore	41,019	73,753	High	ochrobactin	other:other
Region 5.2	redox-cofactor	311,167	333,312			
Region 6.1	RRE-containing	72,350	92,643			
Region 8.1	RiPP-like, terpene-precursor	65,584	96,594	Low	polyhydroxyalkanoate	other:other
Region 10.1	NRPS, T1PKS, hserlactone	200,507	248,567	High	rhizomide A/rhizomide B/rhizomide C	NRPS:Type I
Region 12.1	thioamitides	132,545	159,033			
Region 13.1	terpene	53,977	74,864			
Region 18.1	RiPP-like	62,161	72,967			
Region 33.1	hglE-KS, ranthipeptide	1	24,487			

C

Region	Type	From	To	Similarity Confidence	Most similar known cluster	
Region 4.1	NAPAA	1,092,218	1,126,279			
Region 4.2	terpene-precursor, RiPP-like	1,597,457	1,628,518	Low	polyhydroxyalkanoate	other:other
Region 4.3	thioamitides	1,937,406	1,963,888			
Region 4.4	NRPS, T1PKS, hserlactone	3,595,598	3,643,658	High	rhizomide A/rhizomide B/rhizomide C	NRPS:Type I
Region 4.5	terpene	4,001,278	4,022,165			
Region 4.6	RRE-containing	4,149,984	4,170,277			
Region 8.1	redox-cofactor	115,321	137,466			
Region 8.2	NI-siderophore	374,968	407,702	High	ochrobactin	other:other
Region 8.3	RiPP-like	702,498	713,304			
Region 8.4	RiPP-like	1,056,503	1,067,309			
Region 8.5	hserlactone	1,275,284	1,295,934			

Figure S3. antiSMASH-predicted biosynthetic gene clusters (BGCs) in strains La1 (A), JLW10^T (B), and KB 33 2 (C), showing cluster types, coordinates, and similarity to known MIBiG reference pathways. All three genomes contain NRPS and siderophore clusters, including rhizomide and ochrobactin biosynthesis, alongside several low-similarity orphan BGCs, reflecting shared metabolic potential and ecological adaptation.

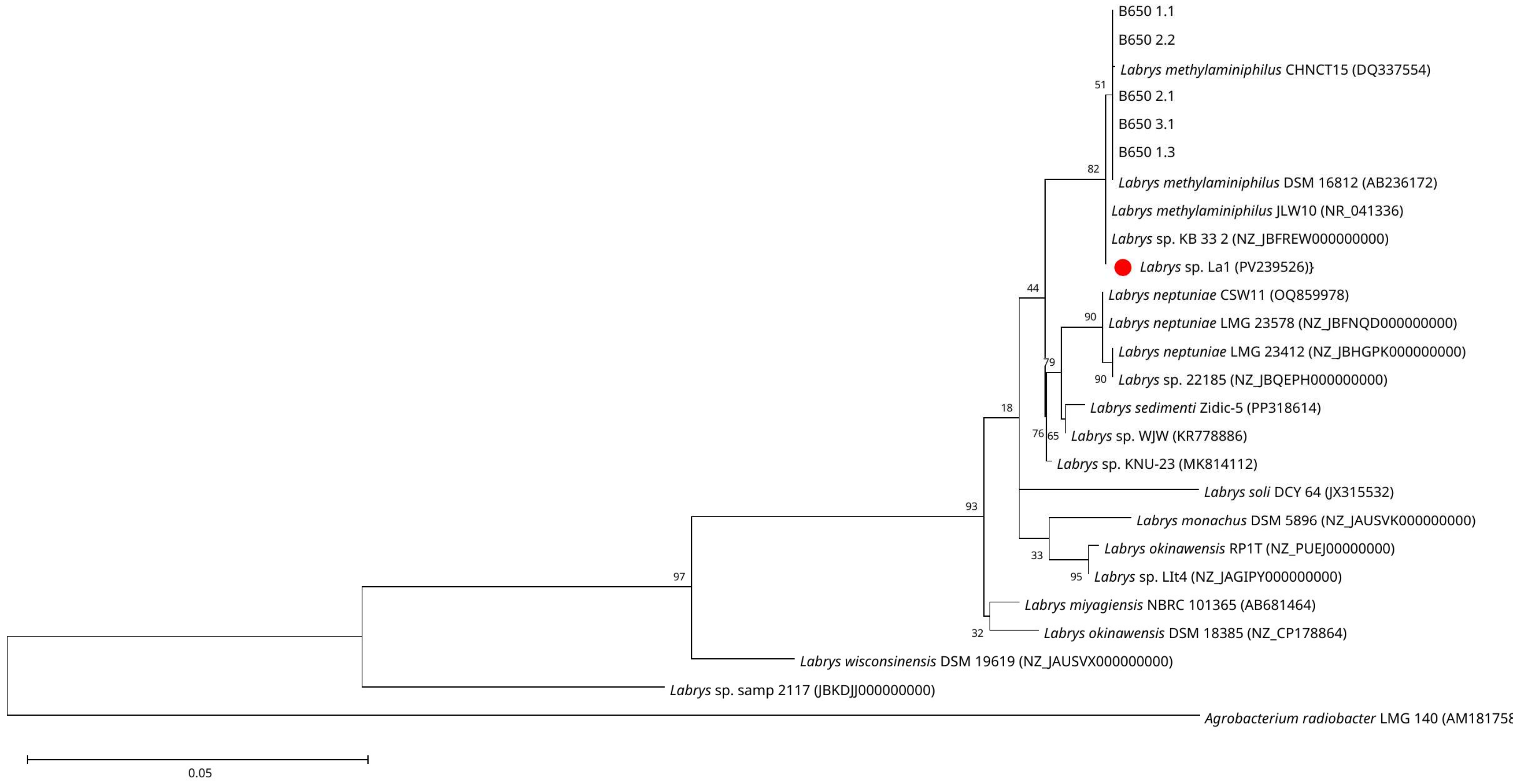

Figure S4. **Phylogenetic analysis of strain La1 and other *Labrys* isolates from *Lupinus luteus* in comparison with representative *Labrys* species**. A maximum-likelihood phylogenetic tree was constructed based on 16S rRNA gene sequences using the TVM substitution model. Bootstrap support values were calculated from 1,000 replicates. *A. radiobacter* LMG 140^{T} was included as the outgroup to root the tree.

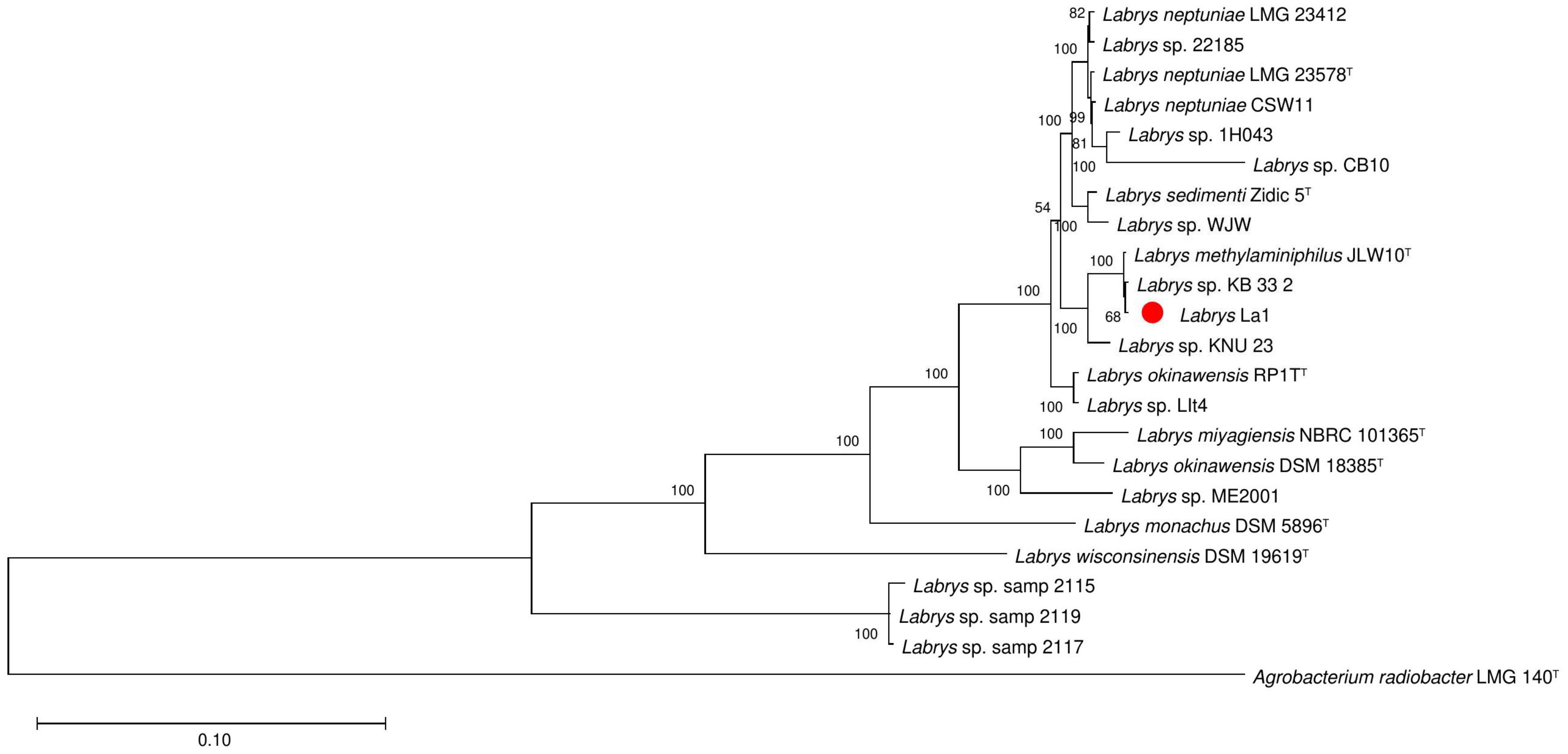

Figure S5. Phylogenomic reconstruction of the genus *Labrys* inferred using the EasyCGTree pipeline based on curated core protein-coding genes. A maximum-likelihood phylogenetic tree based on concatenated alignment of 120 core protein, showing the position of *Labrys* strain La1 (red spot). Bootstrap values were calculated from 1000 replicates to assess branch support. *A. radiobacter* LMG 140^T was included as the outgroup.

Table S1. Quantitative summary of ecological profiling results obtained using Protologger.

Environment	Samples analyzed (n)	Detection frequency (%)	Mean relative abundance (%)	SD
Rhizosphere	1,000	18.30	0.07	0.39
Plant	1,000	13.20	0.14	0.30
Soil	1,000	13.60	0.04	0.07
Wastewater	1,000	11.60	0.01	0.01
Activated sludge	1,000	5.90	0.00	0.01
Freshwater	1,000	3.10	0.01	0.02
Insect gut	1,000	2.10	2.23	4.00
Others (animal/marine)	1,000	≤0.40	≤0.01	≤0.01

**Table S2.** Strain designation and accession numbers of genomes included in the phylogenomic analysis.					
**Species**	**Strain**	**Type Strain**	**Contigs**	**%CG**	**Accesion**
Labrys methylaminiphilus	JLW10	Yes	9	63	2565956506*
Labrys neptuniae	LMG 23578	Yes	49	64	NZ_JBFNQD000000000
Labrys sedimentis	Zidic-5	Yes	109	63.5	NZ_JAXOUF010000000
Labrys okinawensis	RP1	Yes	22	64	NZ_PUEJ01000000
Labrys okinawensis	DSM 18385	Yes	1	62.5	NZ_CP178864
Labrys miyagiensis	NBRC 101365	Yes	122	62.5	NZ_BSPC01000000
Labrys monachus	DSM 5896	Yes	1	66.5	NZ_JAUSVK000000000
Labrys wisconsinensis	DSM 19619	Yes	8	69.5	NZ_JAUSVX000000000
Labrys sp.	La1	No	2	63	NZ_JBMDIH010000000
Labrys sp.	KB 33 2	No	8	63	NZ_JBFREW000000000
Labrys sp.	KNU-23	No	2	63.5	NZ_CP043489
Labrys sp.	CB10b 03	No	2184	65	NJGX00000000‡
Labrys sp.	1H043	No	87	64	JASGFM010000000‡
Labrys neptuniae	CSW11	No	13	64	NZ_JAVSCS010000083
Labrys neptuniae	LMG 23412	No	7	63.5	NZ_JBHGPK010000000
Labrys sp.	22185	No	145	63.5	NZ_JBQEPH010000000
Labrys sp.	WJW	No	140	64	NZ_LYXZ01000000
Labrys sp.	LIt4	No	134	64	NZ_JAGIPY010000000
Labrys sp.	ME2001	No	1079	63	JBIIHI010000000‡
Labrys sp.	2119 meta 259	No	69	66.5	JBKDKQ010000000‡
Labrys sp.	2115 meta 227	No	97	66	JBKDFY010000000‡
Labrys sp.	2117 semi 122	No	157	66	JBKDJJ010000000‡

* Genome sequence obtained from Joint Genome Institute (https://jgi.doe.gov/)
‡ Metagenome-assembled genomes (MAGs)
